# Membrane Permeability and Responsiveness Drive Performance: Linking Structural Features with the Antitumor Effectiveness of Doxorubicin-Loaded Stimuli-Triggered Polymersomes

**DOI:** 10.1021/acs.biomac.4c00282

**Published:** 2024-06-25

**Authors:** Eliézer Jäger, Peter Černoch, Martina Vragovic, Lindomar Jose Calumby Albuquerque, Vladimir Sincari, Tomáš Heizer, Alessandro Jäger, Jan Kučka, Olga Šebestová Janoušková, Ewa Pavlova, Luděk Šefc, Fernando Carlos Giacomelli

**Affiliations:** † Institute of Macromolecular Chemistry, Czech Academy of Sciences, Prague 162 00, Czech Republic; ‡ Centro de Ciências Naturais e Humanas, Universidade Federal do ABC, Santo Andre 09280-560, Brazil; § Center for Advanced Preclinical Imaging (CAPI), First Faculty of Medicine, Charles University, Prague 120 00, Czech Republic

## Abstract

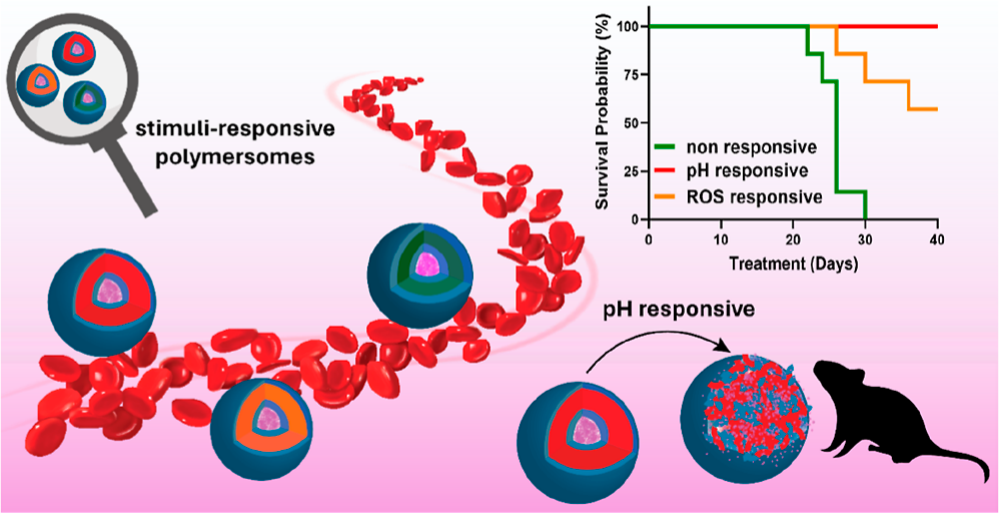

The permeability and responsiveness of polymer membranes are absolutely relevant in the design of polymersomes for cargo delivery. Accordingly, we herein correlate the structural features, permeability, and responsiveness of doxorubicin-loaded (DOX-loaded) nonresponsive and stimuli-responsive polymersomes with their in vitro and in vivo antitumor performance. Polymer vesicles were produced using amphiphilic block copolymers containing a hydrophilic poly[*N*-(2-hydroxypropyl)methacrylamide] (PHPMA) segment linked to poly[*N*-(4-isopropylphenylacetamide)ethyl methacrylate] (PPPhA, nonresponsive block), poly[4-(4,4,5,5-tetra-methyl-1,3,2-dioxaborolan-2-yl)benzyl methacrylate] [PbAPE, reactive oxygen species (ROS)-responsive block], or poly[2-(diisopropylamino)ethyl methacrylate] (PDPA, pH-responsive block). The PDPA-based polymersomes demonstrated outstanding biological performance with antitumor activity notably enhanced compared to their counterparts. We attribute this behavior to a fast-triggered DOX release in acidic tumor environments as induced by pH-responsive polymersome disassembly at pH < 6.8. Possibly, an insufficient ROS concentration in the selected tumor model attenuates the rate of ROS-responsive vesicle degradation, whereas the nonresponsive nature of the PPPhA block remarkably impacts the performance of such potential nanomedicines.

## 1 Introduction

The self-assembly of bilayered nanostructures using lipids, leading to the formation of liposomes, and amphiphilic block copolymers, leading to the formation of polymersomes (PSs), is currently a field of active research. They find applications in the construction of synthetic cells and organelles,^1,2^ catalytic nanoreactors (nanofactories),^3−6^ and nanodevices for biomedical applications.^1,7^ The practical application of liposomes nevertheless suffers from a common drawback, which is a lack of stability.^8^ In this context, the recent progress in polymer chemistry and in controlled polymerization methods gave momentum toward the manufacturing of on-demand block copolymers, allowing for the manufacturing of highly versatile, stimuli-responsive, and temporally stable polymer vesicles.

Particularly considering the manufacturing of cargo-delivery systems, the aqueous lumen of PSs can be used to accommodate therapeutic molecules such as drugs, enzymes, proteins, peptides, and DNA and RNA fragments. Indeed, while stability is certainly a key prerequisite, the permeability and responsiveness of the polymer membranes are equally relevant, and they surely govern the biorelated potential applications of such systems.^9−12^ The polymer membranes are expected to promote a barrier and protect therapeutic agents during systemic circulation, while they are required to regulate the release of the payload at a target location, ideally via a triggered response. Truly, the impermeable and nonresponsive nature of many polymer bilayers remarkably impacts the performance of many potential PS-based nanomedicines.

In this regard, the use of stimuli-responsive PSs which selectively respond to environmental changes may allow for the manipulation of membrane permeability and responsiveness.^13,14^ The clever selection of chemical features and molecular weight of the building units permits the tuning of such highly relevant properties. Particularly focusing on the delivery of chemotherapeutics, pH- and reactive oxygen species (ROS)-responsive polymers can be employed to manipulate the permeability of bilayers and trigger on-demand release of payloads.^15−17^ The pH-responsive vesicles can, in principle, respond to the mildly acidic pH of pathological regions since the acidic extracellular pH is one of the main features of tumors and inflammatory tissues (with pH ranging typically from 6.5 to 7.2 depending on the tumor site and tumor aggressiveness).^18^ The pH-triggered PSs typically have weak acid (carboxylic acid) or weak base (amine) groups that undergo protonation or deprotonation in response to changes in the environmental pH, thereby affecting the integrity of the assemblies.^19,20^ In this framework, poly[2-(diisopropylamino) ethyl methacrylate] (PDPA) exhibits pH-switchable behavior with a p*K*_a(PDPA)_ = 6.8.^21^ Therefore, PDPA has the potential to sense the pH of inflamed tissues, triggering structural alterations and the release dynamics of encapsulated materials in the desired pH window. The use of polymers and self-assemblies responding to reductive environments is also useful from this perspective.^17,22^ The redox potential is significantly different in tumor and normal cells, and it allows, for instance, the use of polymer vesicles containing disulfide bonds, which are susceptible of reduction to thiols in glutathione-rich environments.^23^ More recently, oxidation-sensitive PSs have also been investigated. Due to the production and accumulation of H_2_O_2_ in tumor tissues, the ROS concentration is relatively higher at these sites. The H_2_O_2_ concentration in malignant tumor cells reaches values as high as 100 nM, whereas in normal tissues, the value usually does not exceed 20 nM.^17^ Accordingly, PSs containing boronic ester-based ROS-responsive groups in their composition are potentially able to respond to the environment of tumors and the overexpressed levels of ROS within tumor microenvironments. The H_2_O_2_ can oxidize aryl boronic esters to phenols, leading to hydrophilization of the membranes, thus critically affecting the permeability behavior and ultimately leading to polymer vesicle disassembly.^24,25^

Despite all the efforts made so far, tumor-targeted drug delivery based on stimuli-responsive PSs is still under development, and clinical translation is unclear.^26^ Fundamental investigations concerning the membrane permeability and responsiveness of PSs and their relationship with biological outputs need to be better understood to move forward with confidence. In this study, considering all the above-mentioned issues, we correlate structural features, permeability, and responsiveness with the in vitro and in vivo antitumor performance of nonresponsive and ROS- and pH-responsive PSs. The PDPA-based (pH-responsive) PSs were found to be notably more permeable than the nonresponsive and ROS-responsive assemblies. This is associated with the moderate hydration of the PDPA membrane, given that the working pH (7.4) is close to p*K*_a(PDPA)_ = 6.8, consequently leading to relatively swollen and extended PDPA chains even at pH = 7.4. Additionally, the full protonation of the PDPA chains at pH < 6.8 results in PS disassembly and, consequently, fast payload release. We correlate these specific features with highly relevant biological outcomes as the in vivo antitumor performance of DOX-loaded PDPA-based PSs notably surpasses those of their investigated counterparts.

## 2 Experimental Section

### 2.1 Materials and Chemicals

Sephadex G50, Dulbecco’s phosphate buffered saline (PBS), the dialysis kit Pur-A-Lyzer Maxi-6000 MWCO 6–8 kDa, and Amicon Ultra-4 centrifugal filter units were purchased from Sigma-Aldrich. Doxorubicin hydrochloride salt >99% was purchased from LC Laboratories. Solvents were purchased from Lach:ner (Czech Republic) and dried over molecular sieves (3 Å). The block copolymers poly[*N*-(2-hydroxypropyl)methacrylamide]-*b*-poly[*N*-(4-isopropylphenylacetamide)ethyl methacrylate] (PHPMA_25_-*b*-PPPhA_18_), poly[*N*-(2-hydroxypropyl)methacrylamide]-*b*-poly[4-(4,4,5,5-tetra-methyl-1,3,2-dioxaborolan-2-yl)benzyl methacrylate] (PHPMA_37_-*b*-PbAPE_42_), and poly([*N*-(2-hydroxypropyl)] methacrylamide)-*b*-poly[2-(diisopropylamino)ethyl methacrylate] (PHPMA_29_-*b*-PDPA_74_) were synthesized as previously reported^7,27−29^ with the subscripts referring to the degrees of polymerization of each block determined by ^1^H NMR data.

### 2.2 Preparation of the DOX-Loaded Polymersomes

DOX-loaded PSs were produced using a microfluidic device from Dolomite (Royston, United Kingdom) equipped with a glass micromixer chip with 12 mixing stage microchannels of 50 μm × 125 μm (depth × width). The block copolymers were dissolved in THF/MeOH (80/20 *v*/*v*) at a concentration of 5.0 mg·mL^–1^ as the organic phase (OP). Doxorubicin hydrochloride (1.0 mg) was dissolved in 100 μL of DMSO and added to the OP. The polymer solutions were pumped through the middle channel, and PBS as the aqueous phase (AP) was pumped through the side channels using two independent Dolomite Mitos P-Pumps (Royston, United Kingdom) controlled via computer software. The flow rates were adjustable parameters as follows: for PHPMA_25_-*b*-PPPhA_18_, the flow rates were 200 μL·min^–1^ for AP and 100 μL·min^–1^ for OP; for PHPMA_37_-*b*-PbAPE_42_ and for PHPMA_29_-*b*-PDPA_74_, the flow rates were 100 μL·min^–1^ for AP and 100 μL·min^–1^ for OP. The PSs were collected and passed through a Sephadex G50 column in PBS (pH 7.4) to remove organic solvents and nonencapsulated DOX. The PSs were concentrated to 1.0 mL by using Amicon Ultra-4 centrifugal filter units.

### 2.3 Characterization of the DOX-Loaded Polymersomes

#### 2.3.1 Dynamic Light Scattering

Particle size measurements were conducted by using an ALV/CGS-3 platform-based goniometer system (ALV GmbH). The autocorrelation functions were collected at θ = 90° using the ALV Correlator Control software. The counting time was 120 s, and the distributions of sizes were obtained via the CONTIN analysis with hydrodynamic radius (*R*_H_) calculated using the Stokes–Einstein relation with *D* = τ^–1^*q*^–2^
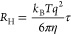
1where *k*_B_ is the Boltzmann constant, *T* is the absolute temperature, *q* is the wave vector, η is the viscosity of the solvent, and τ is the mean relaxation time related to the diffusion of the polymer vesicles. The autocorrelation functions were also analyzed using the cumulant method as^30^
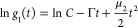
2where *C* is the amplitude of the autocorrelation function, Γ is the relaxation frequency (τ^–1^), and the parameter μ_2_ is known as the second-order cumulant. This approach allowed for the determination of polydispersity indexes (PDI = μ_2_/Γ^2^).

#### 2.3.2 Static Light Scattering

The static light scattering (SLS) measurements were carried out by varying the scattering angle (θ) from 30 to 150° with a 15° stepwise increase. The molecular weight of the PSs (*M*_w(PSs)_) and their radius of gyration (*R*_G_) were determined by using the partial Berry approach as
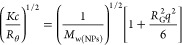
3where the concentration *c* is given in mg·mL^–1^ and *K* is the optical constant, which takes into account the refractive index increments (d*n*/d*c*) determined on a classical Brice–Phoenix differential refractometer.

#### 2.3.3 Electrophoretic Light Scattering

The values of zeta potential (ζ) of the polymer colloids were collected using a Zetasizer Nano-ZS ZEN3600 instrument (Malvern Instruments, UK), which measures the electrophoretic mobility (*U*_E_) and converts it to the value of ζ-potential (mV) through Henry’s equation
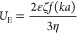
4where ε is the dielectric constant of the medium and η is its viscosity. *f*(*ka*) is Henry’s function calculated through the Smoluchowski approximation (*f*(*ka*) = 1.5).

#### 2.3.4 Cryo-Transmission Electron Microscopy

The cryo-transmission electron microscopy (cryo-TEM) images were captured using a FEI Tecnai G2 Spirit TWIN microscope in bright-field imaging mode with an accelerating voltage of 120 kV. Samples (4 μL) were loaded into electron microscopy grids covered with a holey or lacey carbon supporting film from Electron Microscopy Science. The grids were hydrophilized just before the experiment via glow discharge (Expanded Plasma Cleaner, Harrick Plasma, USA). Excess of the samples was removed by blotting with Whatman no. 1 filter paper, and the grids were rapidly immersed in liquid ethane at −182 °C for vitrification. The vitrified samples were then promptly transferred to the microscope and observed at −173 °C. Image analysis was carried out using ImageJ software.

#### 2.3.5 Small Angle X-ray Scattering

Small angle X-ray scattering (SAXS) profiles were acquired using a pinhole camera (MolMet, Rigaku, Japan, modified by SAXSLAB/Xenocs) connected to a microfocused X-ray beam generator (Rigaku MicroMax 003) operating at 50 kV and 0.6 mA (30 W). Samples were loaded into 2 mm diameter borosilicate capillaries, and the scattering intensity was captured using a Pilatus 300 K detector with an 8 h exposure time. Correction for detector dark noise was applied to the isotropic 2D images, and data reduction was carried out using custom software based on the PyFAI Python library. The resulting *I*(*q*) vs *q* scattering curves were corrected by subtraction of the scattering of the pure solvent. Fitting procedures were conducted using the SASfit software.^31^

### 2.4 DOX Encapsulation and Cumulative Release

The DOX content loaded into the PSs was determined after Sephadex G-50 gel filtration by using UV–vis spectroscopy (λ = 480 nm; ε_480 nm_ = 11,500 cm^–1^ M^–1^) based on an analytical calibration curve. The DOX loading content (LC) and DOX encapsulation efficiency (EE) were determined using the following equations
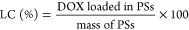
5
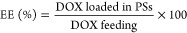
6

The DOX cumulative release experiments were performed by using the dialysis method according to previously published methodologies^7,32^ in four different environmental conditions: PBS (pH 7.4) or acetate buffer (pH 5.3) either in the presence or absence of 1 mM H_2_O_2_. A preswollen cellulose dialysis membrane tube with a molecular weight cutoff (MWCO) of 6–8 kDa (Pur-A-Lyzer) was loaded with 2.0 mL of DOX-loaded PSs at a concentration of 0.5 mg·mL^–1^. Subsequently, the membrane tube was immersed into 3 L of various release media at 37 °C under stirring (350 rpm). The DOX release was monitored at predefined time intervals where 500 μL of the DOX-loaded PSs were sampled from the inner compartment, and the remaining DOX amount was quantified by UV–vis spectroscopy as aforementioned. The sampled amount was further returned to the corresponding membrane tube.

### 2.5 Evaluation of In Vitro Cell Cytotoxicity

#### 2.5.1 Cell Culture

T lymphocyte human Jurkat cells and T lymphocyte mouse EL4 cells (ATCC, Poland) were respectively cultured in RPMI-1640 and Dulbecco’s modified Eagle’s medium (DMEM). The cells were supplemented with 100 units of penicillin and 100 μg·mL^–1^ streptomycin (Life Technologies, Czech Republic) with fetal bovine serum at a concentration of 10% *v*/*v*. They were cultured in 25 cm^2^ flasks at 37 °C with 5% CO_2_.

#### 2.5.2 Evaluation of Cellular Uptake

The uptake of DOX-loaded PSs, in comparison to free DOX (where the DOX content was set to 10 μg·mL^–1^), was quantified through flow cytometry. T lymphocyte cells (1 × 10^5^ cells per well) were seeded in a 24-well plate 1 day prior to incubation. DOX-loaded PSs and free DOX were incubated for 2 h in a 5% CO_2_ environment at 37 °C, and afterward, the cells were centrifuged (1500 rpm) and resuspended in 0.5 mL of PBS with 0.5% *v*/*v* bovine serum albumin (BSA) (this process was repeated two times). Data were collected using a FACS Verse flow cytometer (Becton Dickinson), analyzing 10,000 events per sample, and further processed with the FlowJo software V7.6.1. Mean fluorescence intensity (MFI) values were determined with untreated cells serving as the negative control. All measurements were conducted in triplicate in three independent experiments.

#### 2.5.3 Cell Viability Assay

Cell viability was assessed using the Alamar Blue reagent (Life Technologies, Czech Republic). Cells were plated in 96-well plates (1 × 10^4^ cells per well) and allowed to adhere for 24 h. Subsequently, the cells were exposed to serial dilutions of DOX-loaded PSs, unloaded PSs, and free DOX, added to the medium at a volume of 10 μL, for 72 h of incubation in a 5% CO_2_ atmosphere at 37 °C. Subsequently, 10 μL of Alamar Blue reagent was added to each well and incubated for 4 h in a 5% CO_2_ atmosphere at 37 °C. Resorufin, the active compound of the Alamar Blue reagent, is highly fluorescent only in the presence of viable cells. Fluorescence intensity was measured using a Synergy Neo plate reader (Bio-Tek, Prague, Czech Republic) at λ_ex_ = 570 nm and λ_em_ = 600 nm. The control group consisted of untreated cells. All samples were measured in triplicate in three independent experiments.

### 2.6 Evaluation of In Vivo Antitumor Activity

The in vivo antitumor efficacy of DOX-loaded PSs was investigated in female C57BL/6 black mice with EL4 lymphoma tumors. Six to eight week old C57BL/6 female mice (Anlab, Czech Republic) were employed in tumor shrinkage studies, with *ad libitum* access to food and water. The right flank of the animals was shaved, and subcutaneous injection of T lymphocyte mouse EL4 cells (5 × 10^5^) was performed. After 7 days, mice with established tumors (size 0.15–0.20 cm^3^) were randomly allocated into 5 groups (7–8 animals per group). The treatment consisted of three intravenous injections of 5 mg of DOX (or equivalent)/kg dissolved in 0.9% sodium chloride (saline solution) on days 0, 4, and 8. The control group received a saline solution. Tumor growth and body weight were monitored every 2 days for 30 days. Tumor size, measured with a caliper, was used to compute the respective tumor volume (*V*) as *V* = (*a* × *b*^2^ × π)/6, where *a* is the length and *b* is the width of the tumor surface area. Kaplan–Meier plots were used to represent the survival percentage. The treatment end point was defined as a tumor size of 2 cm^3^ or 40 days of treatment. These experiments were carried out in accordance with The Law of Animal Protection against Cruelty (Act no. 359/2012) of the Czech Republic, and they were conducted under an authority-approved protocol (MSMT-34384/2019-2). The animal house care adhered to the legislation on Experimental Work with Animals (Act no. 246/1992 of the Czech Republic and Decree no. 419/2012), fully complying with European Union directives.

### 2.7 Statistical Analysis

Statistical differences among groups were determined using a two-way ANOVA test. The analyses were conducted using the GraphPad Prism 6 software, and a significance level of *p* < 0.05 was considered statistically meaningful.

## 3 Results and Discussion

### 3.1 Synthesis and Characterization of the Block Copolymers

**Figure 1:**
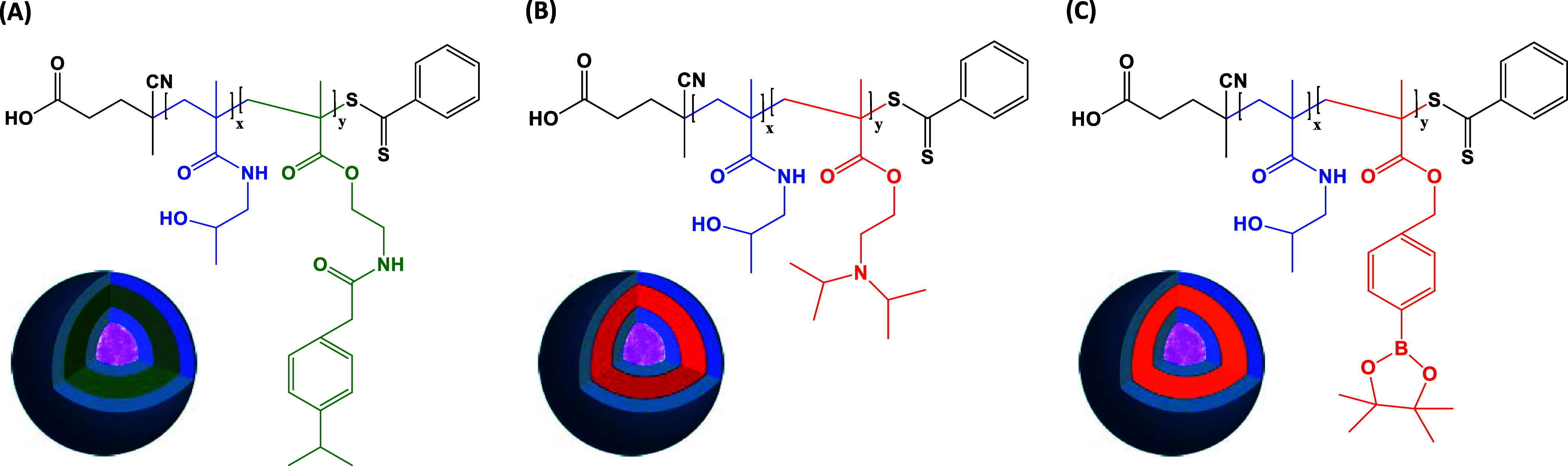
Molecular structure of PHPMA_25_-*b*-PPPhA_18_ (A), PHPMA_29_-*b*-PDPA_74_ (B), and PHPMA_37_-*b*-PbAPE_42_ (C) and respective cartoons depicting the blocks making the inner and outer layers (DOX is represented in pink).

The block copolymers were synthesized by reversible addition–fragmentation chain-transfer polymerization (RAFT). Hydrophilic PHPMA polymer blocks with a modified chain-transfer agent (CTA) were used in the synthetic procedures (PHPMA_25_ mCTA *M*_n_ = 3.1 g·mol^–1^, *D̵* = 1.16, ^1^H NMR = 3575 g·mol^–1^; PHPMA_29_ mCTA *M*_n_ = 3.6 g·mol^–1^, *D̵* = 1.08, ^1^H NMR = 4153 g·mol^–1^; PHPMA_37_ mCTA *M*_n_ = 4.2 g·mol^–1^, *D̵* = 1.12, ^1^H NMR = 5291 g·mol^–1^). The methacrylic monomers, which were nonresponsive, ROS-responsive, and pH-responsive, were subsequently copolymerized using the corresponding PHPMA-mCTA, resulting in nonresponsive, ROS-responsive, and pH-responsive amphiphilic block copolymers. The molecular structures of the block copolymers, which were employed in the preparation of the DOX-loaded PSs, are illustrated in Figure 1, along with a cartoon of the polymer vesicles depicting the respective blocks making the inner and outer layers.

Relevant characteristics of the polymer chains were determined by proton nuclear magnetic resonance (^1^H NMR) and size-exclusion chromatography (SEC). These experimental data are provided, respectively, in Figures S1 and S2 of the Supporting Information. The syntheses of the mCTAs and block copolymers were confirmed by the ^1^H NMR spectra given in Figure S1, and their relatively low degrees of polydispersity (*D̵* < 1.14) were confirmed by the well-defined SEC traces reported in Figure S2. The characteristic signals of protons belonging to the repeating units of the PHPMA-mCTA and the repeating units of the respective hydrophobic blocks are assigned in Figure S1, and they are described in detail elsewhere.^7,27−29,32^ The three block copolymers were manufactured with higher amounts of hydrophobic segments. This has been designed in such a way since PSs were intended to be produced; therefore, an appropriate hydrophilic-to-hydrophobic weight ratio is needed, which thus permits the DOX encapsulation into the aqueous lumen of polymer vesicles. The molecular characteristics of the block copolymers used in this investigation as determined by SEC and ^1^H NMR are given in Table 1.

**Table 1:** Molecular Characteristics of the Block Copolymers Used in This Investigation as Determined by SEC and ^1^H NMR

entry	*M*_n_ (SEC) (g·mol^–^^1^)	*D̵*	*M*_n_ (^1^H NMR) (g·mol^–^^1^)	*M*_n(hydrophobic)_ (g·mol^–^^1^)	*w*_hydrophilic_^e^
PHPMA_29_-*b*-PDPA_74_	20,150^a^	1.04^a^	20,900^b^	14,200^b^	0.29
PHPMA_37_-*b*-PbAPE_42_	21,500^a^	1.13^a^	18,000^c^	12,709^b^	0.29
PHPMA_25_-*b*-PPPhA_18_	18,500^a^	1.10^a^	16,860^d^	9675^d^	0.19

a Determined by SEC in DMF using poly(methyl methacrylate) as a standard.

b Determined by ^1^H NMR in D_2_O/DCl.

c Determined by ^1^H NMR in DMF-*d*_7_.

d Determined by ^1^H NMR in DMF.

e Weight fraction of the hydrophilic block determined based on ^1^H NMR data.

### 3.2 Production and Characterization of DOX-Loaded Polymersomes

**Figure 2:**
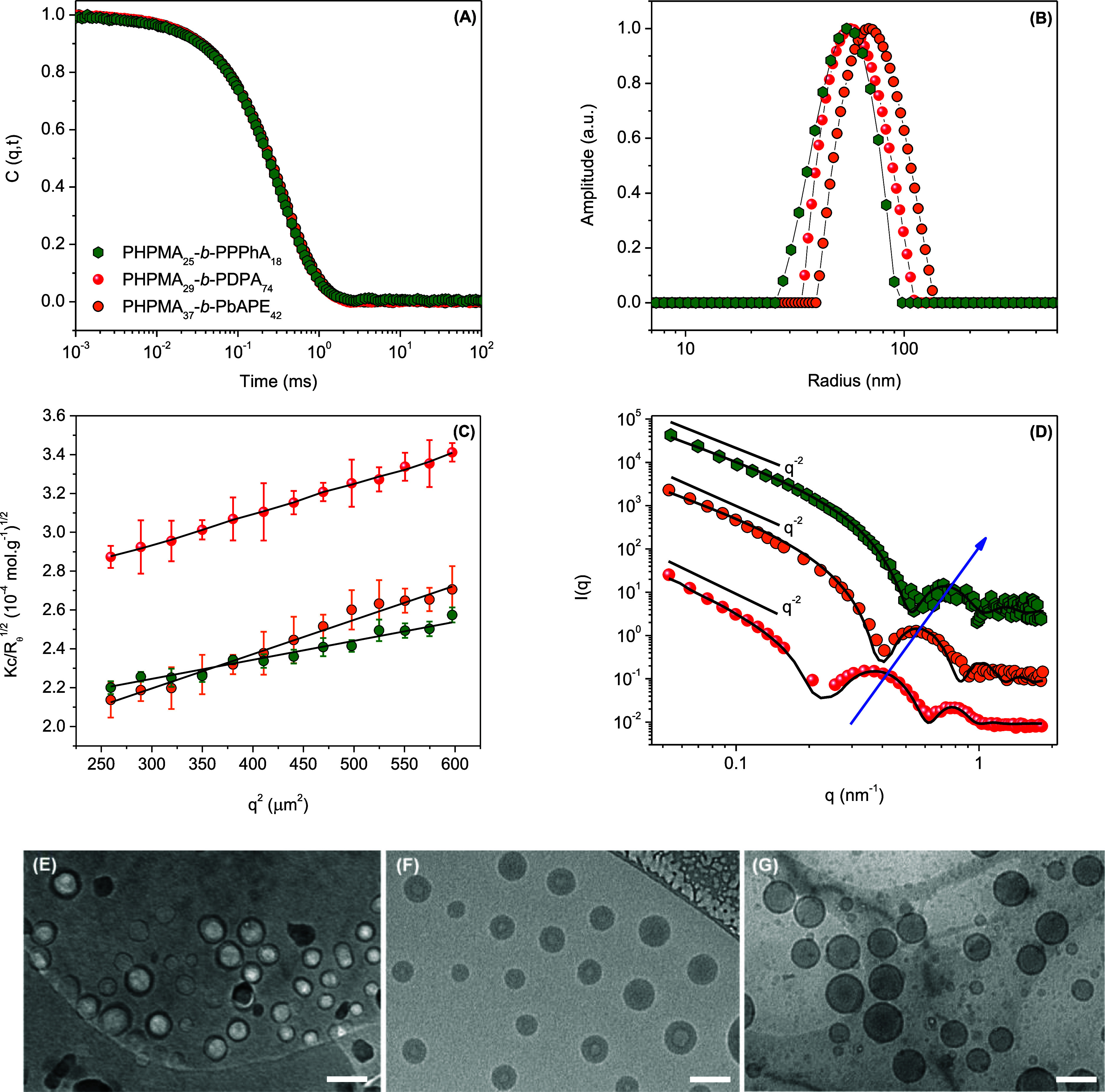
Scattering data acquired for DOX-loaded PSs according to the legend: (A) autocorrelation functions, (B) respective distributions of sizes, (C) SLS (*K*_c_/*R*_θ_^1/2^ vs *q*^2^) data with corresponding linear fittings, and (D) SAXS profiles (symbols) with respective curve fittings (*c*_polymer_ = 1.0 mg mL^–1^ in PBS at pH 7.4). Cryo-TEM images of the self-assemblies produced from PHPMA_29_-*b*-PDPA_74_ (E), PHPMA_25_-*b*-PPPhA_18_ (F), and PHPMA_37_-*b*-PbAPE_42_ (G) (scale bar = 100 nm).

The DOX-loaded PSs were prepared by microfluidics. This methodology enables better control over solvent mixing, therefore leading to the formation of less polydisperse assemblies. Various techniques were employed to characterize the manufactured polymer vesicles, including dynamic light scattering (DLS), static light scattering (SLS), electrophoretic light scattering (ELS), small-angle X-ray scattering (SAXS), and cryo-TEM. The whole set of scattering and imaging data is provided in Figure 2.

The DLS data (Figure 2A,B) point out the formation of relatively small PSs with similar sizes. The PSs produced from PHPMA_37_-*b*-PbAPE_42_ are slightly bigger, as one can check in the quantitative values reported in Table 2. This has indeed relevant consequences on the DOX loading content and encapsulation efficiency, as discussed hereafter.

**Table 2:** Structural Features of the DOX-Loaded Manufactured PSs as Determined by Light Scattering Measurements

entry	*R*_H_ (nm)	PDI	RG/RH	*N*_agg_	ζ (mv)
PHPMA_25_-*b*-PPPhA_18_	50.6	0.12	1.08	1584	–5.6
PHPMA_29_-*b*-PDPA_74_	51.0	0.06	1.02	780	–3.9
PHPMA_37_-*b*-PbAPE_42_	65.0	0.18	1.01	1605	–8.2

The self-assemblies are reasonably homogeneous (PDI < 0.20), which is at least to some extent the result of the employed microfluidic-assisted manufacturing approach. The stability of the produced polymer colloids was found to be notably high, with no signs of nanoparticle aggregation or remarkable increase in nanoparticle size or size dispersity even after three months of manufacturing when stored at 4 °C. The surface charge (ζ-potential values) of the self-assemblies is slightly negative. Since PHPMA shells are nonionizable, one should indeed expect the presence of nearly neutral surfaces. Nevertheless, partial charge partitioning within the polymer shells normally leads to slightly negative surfaces, which are commonly observed for such types of assemblies.^33,34^

The SLS measurements (Figure 2C) were acquired to determine the number of aggregation (*N*_agg_ = *M*_w(PSs)_/*M*_w(polymer by SEC)_) and radius of gyration (*R*_G_) of the assemblies (Table 2). Since PSs are hollow spheres filled with solvent, the amphiphilic membranes, which concentrate the scattering mass of the assemblies, are located at the surface of a sphere. Therefore, *R*_G_/*R*_H_ = 1 is typically assigned for hollow spheres.^35^ The theoretical value for homogeneous compact hard spheres is 0.774.^36^ Respectively, the data reported in Table 2 suggest the block copolymer self-assembly into PSs. The values of *N*_agg_ are fairly high, thus also suggesting the formation of PSs.^37^ These assumptions are confirmed by the acquired SAXS profiles and cryo-TEM images, which are also provided in Figure 2. The SAXS data enabled quantitative assessment of internal dimensions (membrane features). The experimental data reported in Figure 2D underline for the whole set of assemblies an upward profile in the low-*q* range. This follows a *q*^–2^ power-law dependence, which is typically assigned to the presence of polymer vesicles. Accordingly, the oscillations observed in the *q* range ∼0.9–1.0 nm^–1^ are associated with the membrane characteristics. The scattering profiles were fitted using the bilayered vesicle form factor with the radius of the inner compartment (*R*_c_), the thickness of the hydrophilic outer shell (*t*_h_), and the hydrophobic inner segment (*t*_t_) as adjustable parameters along with the respective electron densities. The fitted parameters are given in Table 3. Since the *q*^–2^ slope with no oscillations at small *q*-values has been monitored, a large polydispersity for *R*_c_ was needed to properly fit the experimental data. This particular parameter accordingly holds a high degree of uncertainty, and therefore, it has been omitted.

**Table 3:** Structural Parameters of the DOX-Loaded PSs as Determined by SAXS Measurements and Respective Curve Fittings (All Dimensions Are Given in nm)^a^

entry	*t*_t_	*t*_h_	*w*
PHPMA_25_-b-PPPhA_18_	3.6	4.1	11.8
PHPMA_29_-*b*-PDPA_74_	10.7	4.5	19.7
PHPMA_37_-*b*-PbAPE_42_	8.0	3.4	14.8

a Wall thickness (*w*): *t*_h_ + *t*_t_ + *t*_h_.

The fitting procedures using the bilayer form factor evidenced a smaller wall thickness for PHPMA_25_-*b*-PPPhA_18_. This is compatible with the shoulders in the SAXS profiles, respectively, located at a higher *q*-range for the nonresponsive self-assemblies. The whole set of scattering measurements indeed agrees with the cryo-TEM images, where one can easily notice spherical particles with high transmission in their centers, thus robustly confirming the vesicular morphology.

The responsiveness to external stimuli of the produced PHPMA_29_-*b*-PDPA_74_ and PHPMA_37_-*b*-PbAPE_42_ polymer vesicles has been evaluated at acidic pH and in the presence of ROS species. The remarkable light scattering reduction at acidic pH for PHPMA_29_-*b*-PDPA_74_ suggests fast macromolecular disassembly. As for the case of PHPMA_37_-*b*-PbAPE_42_, light scattering reduction in the presence of H_2_O_2_ is slower; however, it also implies a response to external stimuli and a ROS-induced self-immolative degradation of the PSs.^28^ These data are provided in the Supporting Information (Figure S3).

### 3.3 Evaluation of DOX Encapsulation and Release Profiles

**Figure 3:**
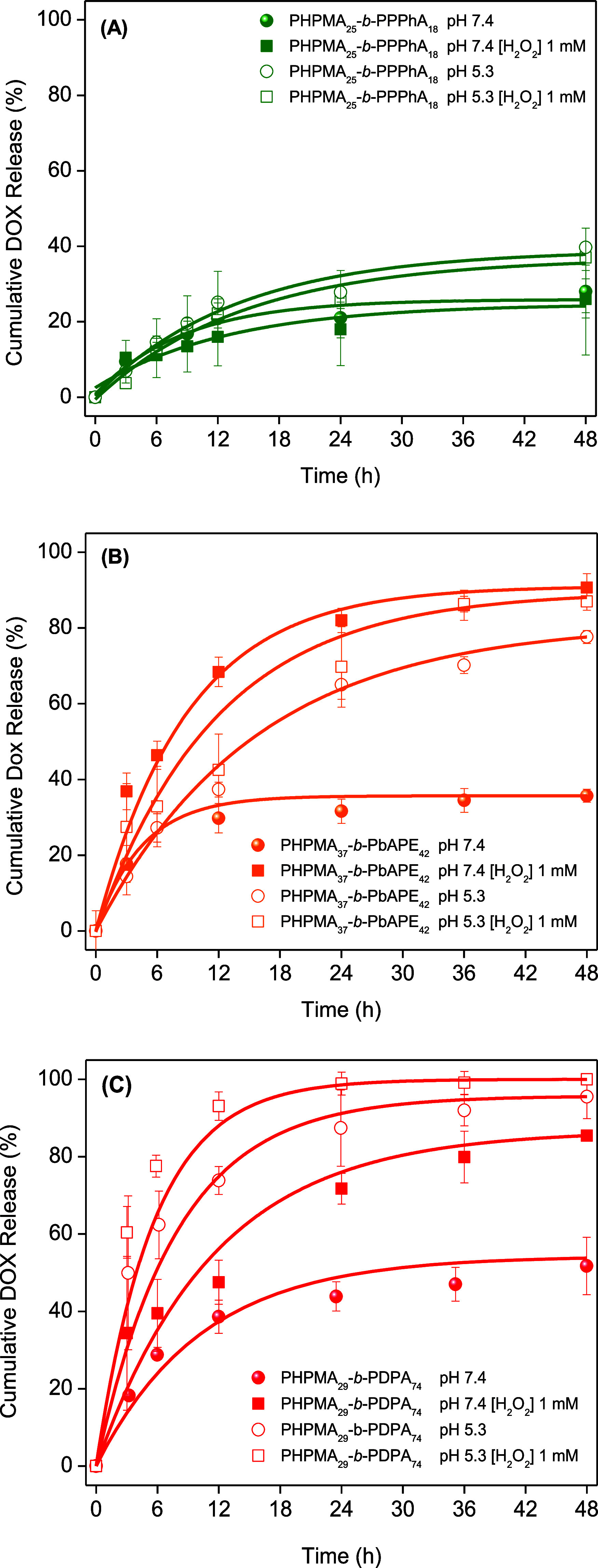
Cumulative DOX release from PHPMA_25_-*b*-PPPhA_18_ (A), PHPMA_37_-*b*-PbAPE_42_ (B), and PHPMA_29_-*b*-PDPA_74_ (C) PSs at different environmental conditions according to the legends (*n* = 3).

The PS DOX release profiles under various environmental conditions are listed in Figure 3. We portray the DOX cumulative release as a function of time for the drug-loaded PSs at different pH values and in the presence or absence of H_2_O_2_ at 1 mM. The values of DOX loading content and encapsulation efficiency are reported in Table 4.

**Table 4:** Parameters for DOX Release Data Reported in Figure 3 Were Obtained by Fitting the Experimental Profiles Using eq 7

entry	pH	[H_2_O_2_] mM	τ (h)	CR_max_ (%)	*R*^2^	EE (%)	LC (%)
PHPMA_25_-*b*-PPPhA_18_	7.4		9 ± 2	26 ± 2	0.94	11.8	2.4
	5.3		14 ± 3	39 ± 3	0.96	11.8	2.4
	7.4	1	13 ± 5	25 ± 3	0.88	11.8	2.4
	5.3	1	15 ± 4	37 ± 4	0.95	11.8	2.4
PHPMA_29_-*b*-PDPA_74_	7.4		10 ± 1	54 ± 2	0.89	20.9	4.2
	5.3		7.9 ± 0.8	96 ± 3	0.92	20.9	4.2
	7.4	1	11.5 ± 0.9	87 ± 4	0.91	20.9	4.2
	5.3	1	5.7 ± 0.7	100 ± 6	0.89	20.9	4.2
PHPMA_37_-*b*-PbAPE_42_	7.4		4.5 ± 0.8	36 ± 2	0.94	29.8	6.0
	5.3		16 ± 1	81 ± 2	0.96	29.8	6.0
	7.4	1	8.9 ± 0.9	91 ± 3	0.88	29.8	6.0
	5.3	1	12 ± 2	90 ± 2	0.98	29.8	6.0

The values of LC fall within the range of 2.4–6.0%. These values possibly reflect the self-assembly behavior of the PSs, respectively, disparate volumes of the aqueous lumen, and the number of aggregation, which accordingly reflect the overall number of PSs per volume unit produced. The drug and polymer feeding were kept fixed during the preparation of the DOX-loaded PSs; nevertheless, different values of *N*_agg_ have been determined (Table 2). The value for PHPMA_25_-*b*-PPPhA_18_ is relatively high (1584); therefore, a small number of particles is present in the system compared to PHPMA_29_-*b*-PDPA_74_, for instance, thus resulting in smaller values of LC and EE. Although the value of *N*_agg_ is similar for PHPMA_37_-*b*-PbAPE_42_ (*N*_agg_ = 1605), the values of LC and EE are higher (roughly 6 and 30%, respectively) compared to those of the other assemblies. This is possibly the result of the larger particles present. Although the difference in *R*_H_ is not remarkable (ranging from 50 to 65 nm, approximately), one has to take into account that the volume scales with *R*^3^, meaning that such a difference more than doubles the particle volume, therefore justifying at least to some extent the higher values of LC and EE calculated. We nevertheless underscore that the biological evaluations discussed hereafter were conducted by keeping the DOX amount fixed.

The DOX release profiles highlight fast DOX release at the first 12 h, and afterward, the rate of elution progressively reduces, although drug release continues up to the end of the time scale of the experiment (48 h). The evidenced nonlinear DOX release profiles could be mathematically fitted by using exponential growth as
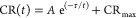
7

This approach, instead of using several different models for the same experimental data, provides outputs that can be compared among the different polymer vesicles. The fitting procedure gives CR_max_, τ, and A as fitted parameters, which are the maximum release, characteristic time, and an offset, respectively. The adjustable parameters are all listed in Table 4. The values of *R*^2^ confirm that the fitting approach is able to properly describe experimental release profiles since they are always higher than 0.87. The characteristic time is a quantitative value related to the diffusion of the probe toward the polymer membranes.

The profiles reported in Figure 3 highlight the low permeability of the membranes produced using PHPMA_25_-*b*-PPPhA_18_ regardless of the environmental conditions since maximum DOX elution (CR_max_) was never higher than 40% at the end of the experiment. The nonresponsive PHPMA_25_-*b*-PPPhA_18_ polymer vesicles possibly undergo noncovalent π–π stacking interaction (orbital overlap) between the π bonds of the aromatic rings, thus restricting chain mobility and leading to a fairly robust barrier property. Similar behavior has been previously evidenced during the elution of rhodamine B.^29^ The released amount may also be linked to a fraction of DOX molecules that were possibly not fully loaded into the aqueous lumen of the assemblies but rather adsorbed at their outer surfaces. The environmental pH or the presence of H_2_O_2_ in the medium does not remarkably change the elution profiles, although the acidification of the media leads to slightly higher values of CR_max_. Since DOX is a weak base, its solubility in aqueous media changes depending on the environmental conditions. The nonencapsulated DOX amount may be faster desorbed at acidic pH or in the presence of H_2_O_2_, regardless of the permeability feature of the polymer vesicles.

The permeability and responsiveness of the PDPA membranes, on the other hand, lead to a notably distinct behavior. Indeed, such a membrane is the most permeable to DOX at pH 7.4 and in the absence of H_2_O_2_. The membranes produced by using PDPA exhibit a significantly greater thickness in comparison to their nonresponsive counterpart. However, they impart a considerably higher level of permeability. The enhanced permeability of the PDPA-based vesicles is likely associated with the chemical characteristics of the polymer chains and their packing density within the membranes rather than being determined by the degree of polymerization of the hydrophobic block and its length. This is indeed linked to the pH-responsive behavior of the PDPA block. Since p*K*_a(PDPA)_ = 6.8, a non-negligible fraction of protonated species is still present at pH = 7.4 (approximately 20% of the amino groups are protonated at this environmental condition),^38^ leading to the presence of water-swollen hydrophobic segments and extended polymer chains within the membrane, therefore resulting in a leaky feature and allowing the diffusion of the therapeutic agent through the polymer wall. The DOX elution is also dependent on the pH of the medium and the presence of H_2_O_2_. The value of C*R*_max_ = 54% at pH 7.4 and in the absence of H_2_O_2_ reaches values over 90% when the medium is acidified. Furthermore, the characteristic elution time (τ) is simultaneously reduced, meaning that the drug is also eluting faster as pH reduces. This is the result of a triggered payload release since, at pH 5.3, the PDPA chains are fully protonated, leading to PS disassembly. On the other hand, the DOX release is attenuated when the therapeutic agent is loaded into the ROS-responsive PHPMA_37_-*b*-PbAPE_42_ vesicles, thus underlining the low permeability feature of the PbAPE_42_ membrane at pH 7.4 and in the absence of H_2_O_2_, although a small fraction is released, possibly also due to a fraction of DOX molecules that was physically adsorbed at the outer surface rather than encapsulated in the aqueous lumen of the assemblies. The values of CR_max_ are nevertheless over 80% in the presence of H_2_O_2_. The acidification of the medium also augments DOX release. The pH-dependent behavior in such a case might also be assigned to changes in the solubility of the therapeutic agent. DOX has p*K*_aDOX_ = 9.9,^39^ and the acidification of the environment enhances its water solubility, resulting in more hydrophilic molecules. Truly, regardless of pH responsiveness, DOX seems to be faster released at an acidic pH. Such disparate DOX release features and triggered payload release in an acidic environment have a notable impact, particularly on the in vivo performance of the DOX-loaded polymer vesicles, as discussed hereafter.

### 3.4 Biological Evaluations

#### 3.4.1 In Vitro Cellular Uptake and Cell Cytotoxicity

**Figure 4:**
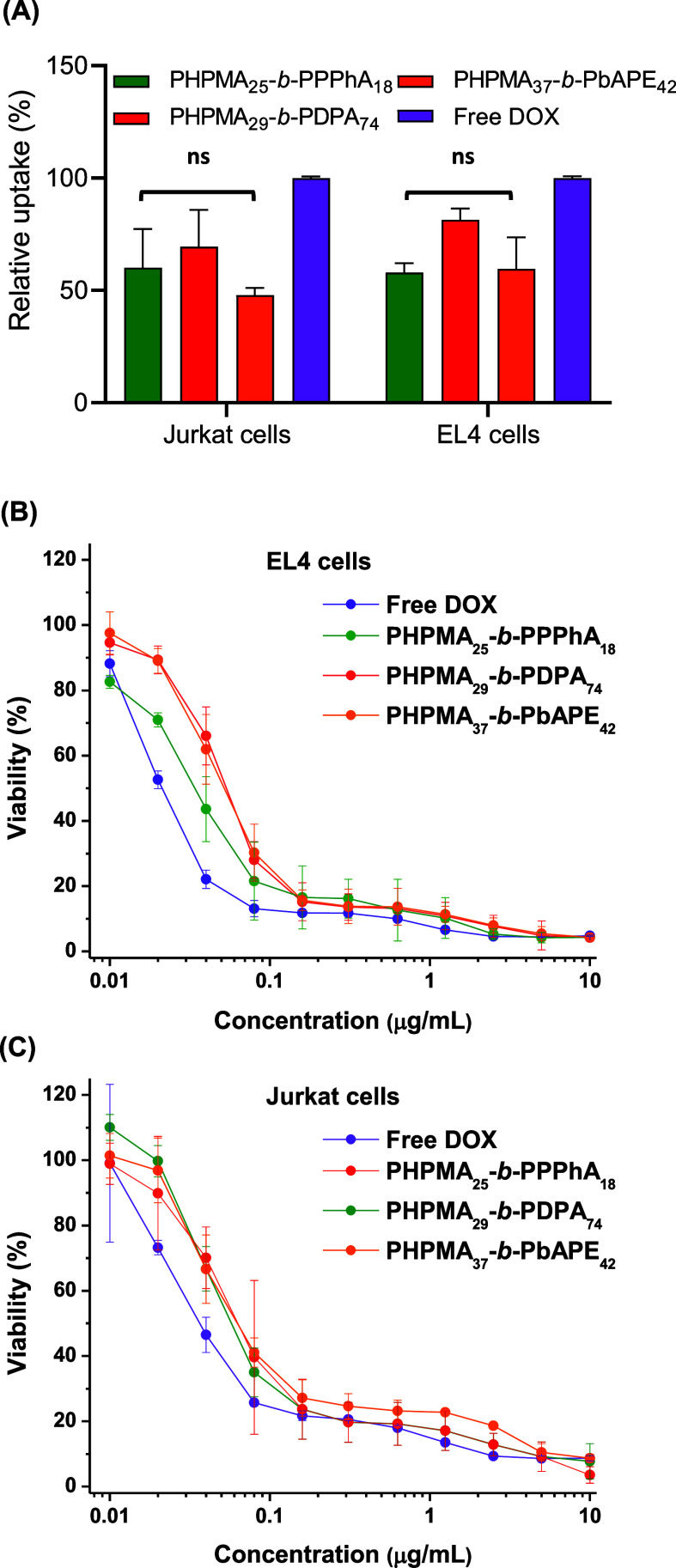
DOX uptake by Jurkat and EL4 cells after 2 h of incubation (*n* = 3) (A). Viability of EL4 (B) and Jurkat (C) cells after 72 h of incubation with different concentrations of free DOX or DOX loaded into PSs according to the legend (*n* = 3). Only nonsignificant (ns) relative to free DOX are depicted for clarity (*p* < 0.05).

The evaluation of cellular uptake and cell cytotoxicity of the produced DOX-loaded PSs was conducted by putting them in contact with EL4 lymphoma and Jurkat cells. The comparative analyses were performed with the results obtained from free DOX administered in equivalent quantities. Figure 4A depicts the MFI values derived from the DOX cellular uptake. The intrinsic fluorescence intensity of DOX proves to be a valuable metric for evaluating its cell internalization. The results indicate that the quantity of DOX taken up by the cells is comparable, regardless of its encapsulation or not. However, it is important to note that only free DOX is likely to be internalized by the cells through a diffusion pathway, while DOX-loaded PSs must undergo internalization through endocytosis due to their large size. The cell uptake data are to some extent expected considering the similar size, surface charge, and surface coating of the polymer vesicles (Figure 2, Table 2).

The in vitro anticancer performance of the DOX-loaded PSs was evaluated in both cell lines (Figure 4B,C). The values of IC_50_ for Jurkat cells are 0.036 for free DOX, 0.057 for PHPMA_25_-*b*-PPPhA_18_, 0.064 for PHPMA_29_-*b*-PDPA_74_, and 0.064 μg·mL^–1^ for PHPMA_37_-*b*-PbAPE_42_. The respective values are 0.021, 0.054, 0.034, and 0.051 μg·mL^–1^ for EL4 cells. The comparable values affirm the effective delivery of DOX to both cell lines, resulting in significant cytotoxic effects. The slightly lower IC_50_ values for free DOX can be attributed to its capacity to diffuse across the cell membranes. Nevertheless, similar results are reported for DOX-loaded PSs. In in vitro assays, the extracellular medium is not as acidic as it is known to be in in vivo tumor microenvironments.^18^ Therefore, the DOX-loaded vesicles must be endocytosed, regardless of the responsiveness, to be effectively delivered to the intracellular compartment. Consequently, the effect of the stimulus has been only marginally evidenced.

#### 3.4.2 Evaluation of In Vivo Antitumor Activity

**Figure 5:**
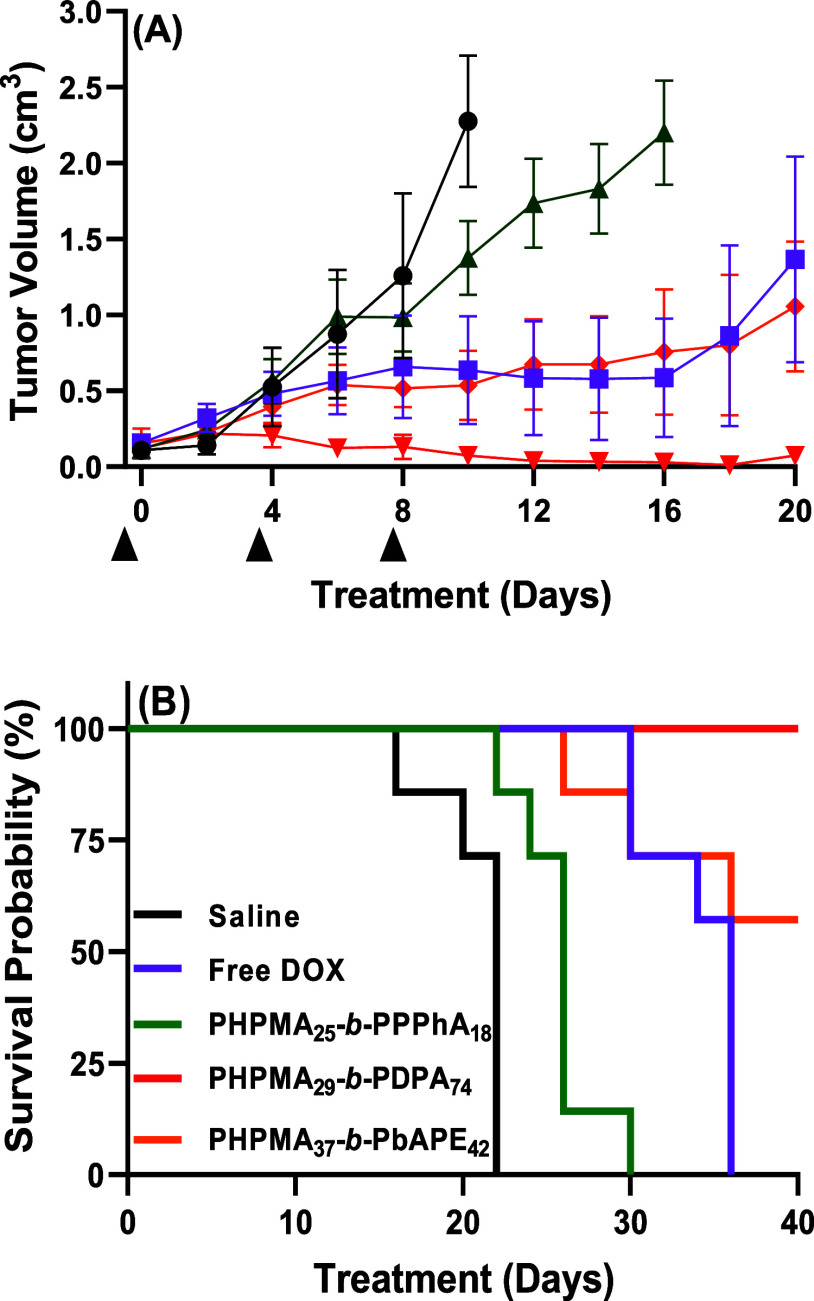
Tumor volume in cm^3^ (A) and Kaplan–Meier survival plot (B) as a function of time for mice treated with saline, free DOX, and DOX-loaded PSs at 5 mg·kg^–1^ DOX or equivalent according to the legend (the data are given as the mean ± SD; *n* = 7–8 mice). The arrows indicate the days of DOX or equivalent administrations.

In the step further, the in vivo therapeutic effect of the different DOX-loaded PSs was evaluated in subcutaneous EL4 (mouse lymphoma) tumor-bearing C57BL/6N mice. Seven days after tumor inoculation, saline solution, free DOX, and equivalent DOX amounts loaded into PSs were administered intravenously in three doses of 5 mg·kg^–1^ with 4 day intervals. Figure 5 provides data of the tumor volume (A) and survival rate (B) as a function of time. The experimental results highlight disparate performance depending on the nanocarrier. The EL4 lymphoma is notably aggressive, and rapid tumor growth is observed when a saline solution is administered (Figure 5A, black circles). This leads to the death of the entire control group within 22 days (Figure 5B, black line). The treatment with DOX-loaded PHPMA_25_-*b*-PPPhA_18_ PSs is not effective in reducing tumor volume or increasing the survival rate since the death of the entire group occurred within a shorter period of time compared with the administration of free DOX. This behavior is most probably linked to the restricted permeability of the nonresponsive polymer membrane, which accordingly attenuates the therapeutic action of DOX at the tumor site.

The tumor volumes after administration of free DOX and DOX-loaded PHPMA_37_-*b*-PbAPE_42_ PSs are, to some extent, similar within experimental errors. Indeed, DOX has a short half-life (30 min to 3 h),^40,41^ leading to relatively fast drug clearance. Yet, DOX treatment extends the survival time from 14 to 20 days to a total of 36 days (Figure 5B). The survival rate is not as high as after the administration of DOX-loaded PHPMA_37_-*b*-PbAPE_42_ PSs, likely due to the cardiotoxic effects of the free drug.^42,43^

On the other hand, the administration of DOX-loaded PHPMA_29_-*b*-PDPA_74_ leads to a remarkable reduction in tumor volume. Although the tumor volume initially experiences a slight increase, it is less pronounced than after other administrations and begins to decrease after the second administration. Significantly, the survival rate remains at 100%, with no deaths observed in the treated group within the time frame of the experiment (tumor size less than 2 cm^3^ or 40 days). This underscores the exceptional effectiveness of the pH-responsive PSs loaded with DOX in suppressing tumor growth and prolonging mouse survival. Taking all of the biological assays together, one can notice that the cytotoxicity of the nanomedicines in vitro does not properly correlate with their capacity to inhibit tumor growth in vivo. The absence of correlation between in vitro and in vivo data has also been documented by others.^44^ Indeed, in vitro conditions frequently fail to replicate factors inherent in the in vivo setting such as complex protein and cellular interactions, physiological barriers, and the specific microenvironment of the targeted sites.

Overall, these experimental data underline that permeability and responsiveness are important features to be evaluated when designing cargo delivery systems based on polymer vesicles. We herein highlight that the pH-responsive behavior of the PDPA chains enables triggered and fast DOX release in slightly acidic environments and reasonably sustained release under physiologically buffered conditions, thus allowing for highly effective antitumor activity. We speculate that the less effective performance of ROS-responsive assemblies is due to an insufficient ROS concentration in the selected tumor model, therefore restricting or at least attenuating the rate of ROS-responsive vesicle disassembly in the tumor environment. The low permeability and nonresponsive nature of the PPPhA_18_ block notably impact drug release and, consequently, the biological outputs.

## 4 Conclusions

In this study, we evaluated the structural features, permeability, and responsiveness of disparate PSs and correlated their properties with their in vitro and in vivo antitumor performance. Nonresponsive, pH-responsive, and ROS-responsive DOX-loaded PSs were successfully produced by using block copolymers with distinct chemical natures. They were further detailed characterized with regard to their structure, DOX loading, and release profile. The experimental data underlined that pH-responsive PDPA-based assemblies are notably more permeable than their nonresponsive or ROS-responsive counterparts, and DOX is quickly released in acidic media as driven by PS disassembly. Such stimuli-responsive features impart to them outstanding biological performance with in vivo antitumor activity notably improved. Possibly, an insufficient ROS concentration in the selected tumor model restricts or at least attenuates the rate of ROS-responsive PS disassembly. The nonresponsive PPPhA block is notably less permeable than its counterparts, consequently restricting drug release and the performance of such potential nanomedicines.

## Supporting Information Available

^1^H NMR spectra and GPC traces for PHPMA_m_-mCTA and the diblock copolymers used in the investigation and light scattering intensity as a function of time for PHPMA_25_-*b*-PbAPE_42_ and PHPMA_29_-*b*-PDPA_74_ PSs under different stimuli (PDF)
