# Supplementary material for: Membrane Permeability and Responsiveness Drive Performance: Linking Structural Features with the Antitumor Effectiveness of Doxorubicin-Loaded Stimuli-Triggered Polymersomes
Source: Biomacromolecules. 2024 Jun 25;25(7):4192–202. doi: 10.1021/acs.biomac.4c00282 (PMC11238342; doi:10.1021/acs.biomac.4c00282)
Supplement: Supplementary file 1 — bm4c00282_si_001.pdf [file bm4c00282_si_001.pdf]

# Supplementary File

## Membrane Permeability and Responsiveness Drive Performance: Linking Structural Features with the Antitumor Effectiveness of Doxorubicin-Loaded Stimuli-Triggered Polymersomes

*Eliézer Jäger,[1,*] Peter Černoch,[1] Martina Vragovic,[1] Lindomar Jose Calumby Albuquerque,[1,2,§] Vladimir Sincari,[1] Tomáš Heizer,[3] Alessandro Jäger,[1] Jan Kučka,[1] Olga Šebestová Janoušková,[1ϕ] Ewa Pavlova,[1] Luděk Šefc,[3] Fernando Carlos Giacomelli[2,*]*

[1] Institute of Macromolecular Chemistry, Czech Academy of Sciences, Prague, 162 00 Czech Republic

[2] Centro de Ciências Naturais e Humanas, Universidade Federal do ABC, Santo André, 09280-560 Brazil

[3] Center for Advanced Preclinical Imaging (CAPI), First Faculty of Medicine, Charles University, Prague, 120 00 Czech Republic

[§] Current address: Brazilian Synchrotron Light Laboratory (LNLS), Brazilian Center for Research in Energy and Materials (CNPEM), Campinas, 13083-100 Brazil

[ϕ] Current address: Faculty of Science, Centre of Nanomaterials and Biotechnology, Jan Evangelista Purkyně University in Ústí nad Labem, Ústí nad Labem, 400 96 Czech Republic

*Corresponding Authors:

Fernando Carlos Giacomelli - e-mail: fernando.giacomelli@ufabc.edu.br

Eliézer Jäger - e-mail: jager@imc.cas.cz

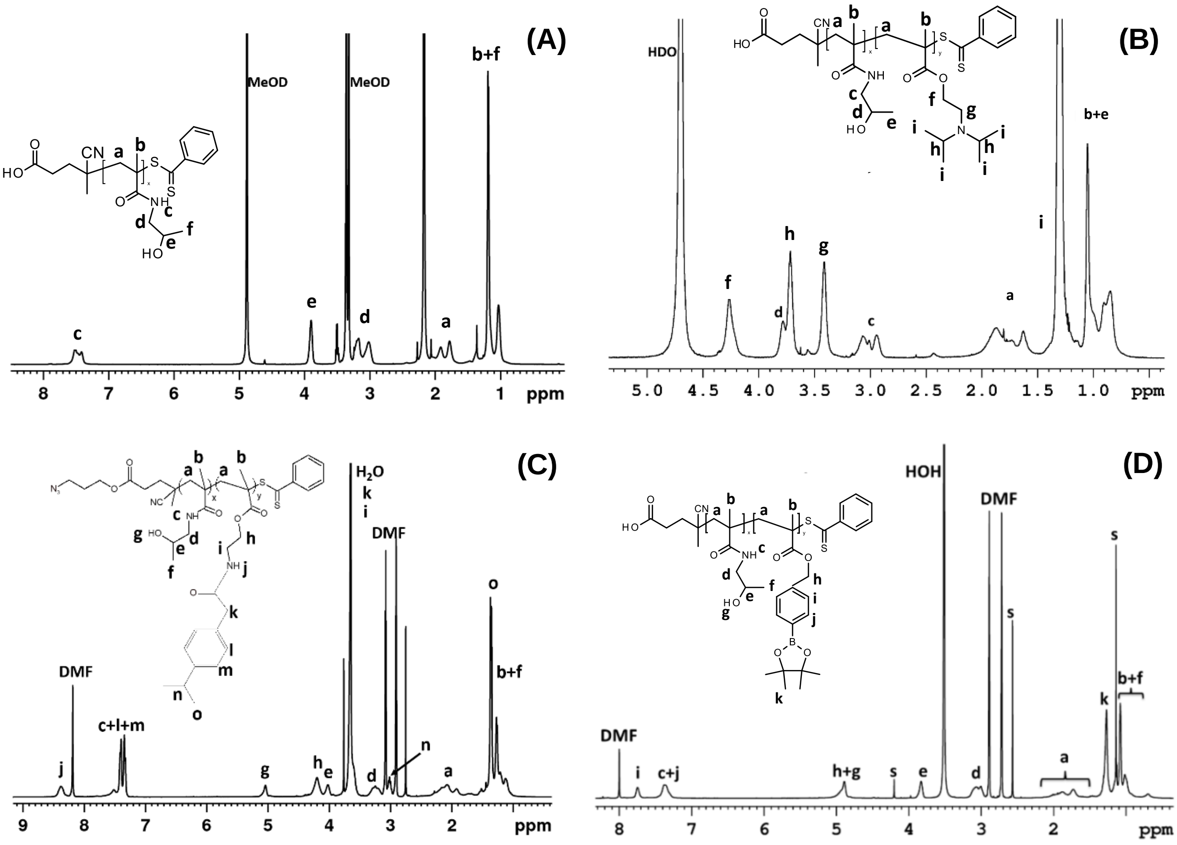


**Figure S1**. $^{1}$H NMR spectrum of $PHPMA_{29}$ macroCTA measured in MeOH-$d_4$ (A) and of $PHPMA_{29}$-*b*-$PDPA_{74}$ measured in $D_2O$/DCl (B). $^{1}$H NMR spectrum of $PHPMA_{25}$-*b*-$PPPhA_{18}$ (C) and of $PHPMA_{37}$-*b*-$PbAPE_{42}$ measured in $d_7$-DMF (D).

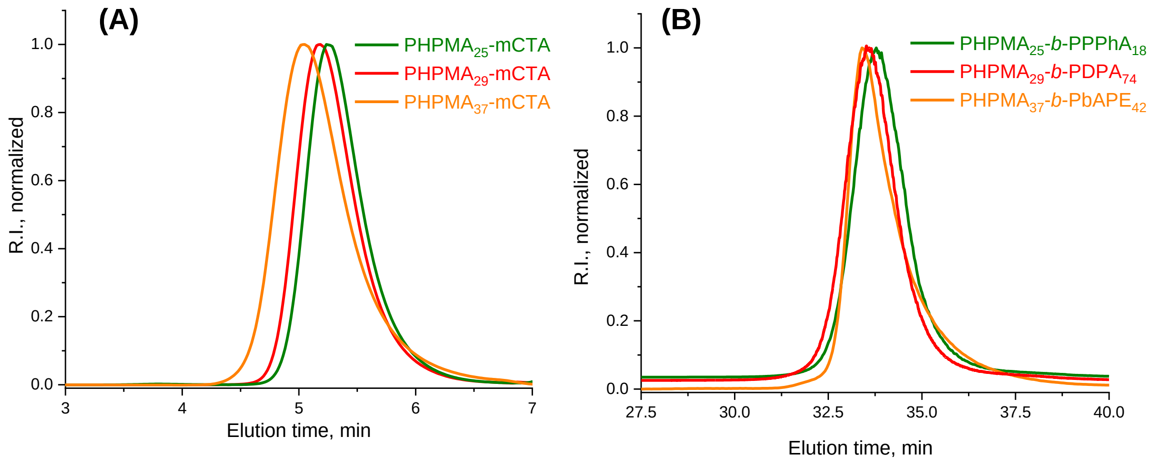


**Figure S2**. SEC traces of PHPMA25-mCTA, PHPMA29-mCTA and PHPMA37-mCTA (A) in MeOH/acetate buffer pH 6.5 (80/20 *v/v*) and for PHPMA25-*b*-NR18, PHPMA29-*b*-pH74 and PHPMA37-*b*-ROS42 block copolymers (B) in DMF.

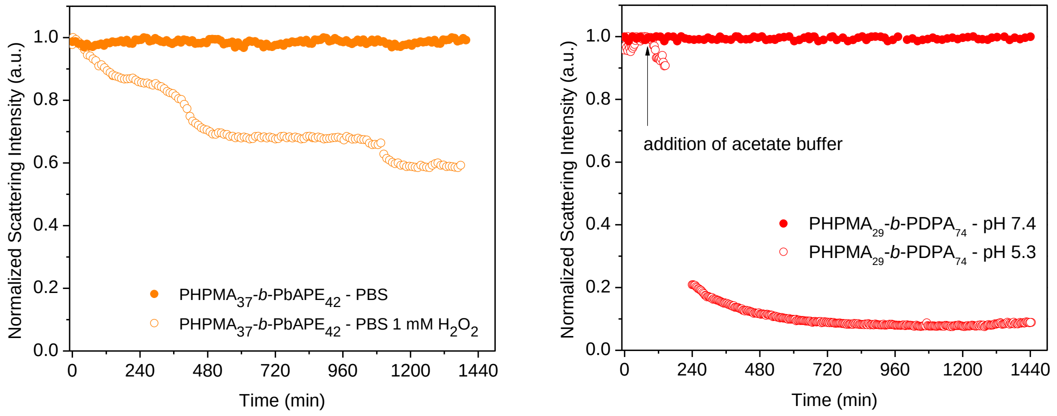


**Figure S3**. Normalized light scattering intensity as a function of time for PHPMA25-*b*-PbAPE42 (left) and PHPMA29-*b*-PDPA74 (right) polymersomes respectively in PBS pH 7.4 and the presence of 1 mM $H_2O_2$, and in PBS at pH 7.4 and acidic media (pH 5.3) according to the legends.
